# Incidence and factors associated with the recurrence of Rathke's cleft cyst after surgery: A systematic review and meta-analysis

**DOI:** 10.3389/fsurg.2022.1065316

**Published:** 2023-01-05

**Authors:** Ao Qian, Jing Zhou, Xin Zhang, Jiaojiao Yu, Xiaoshu Wang

**Affiliations:** Department of Neurosurgery, The First Affiliated Hospital of Chongqing Medical University, Chongqing, China

**Keywords:** rathke’s cleft cyst, recurrent incidence, recurrent factor, systematic review, meta-analysis

## Abstract

**Backgroud:**

Recurrence of Rathke's cleft cyst (RCC) is not uncommon after surgery, and the associated factors and incidence of relapse deserve a systematic summary.

**Methods:**

This study was conducted in accordance with Preferred Reporting Items for Systematic Review and Meta-Analysis (PRISMA) guidelines. The Pubmed, Embase, Cochrane, and Web of Science databases were searched until September 12, 2022. Studies with significant results of recurrent factors or specific incidences of RCC recurrence and mean/median follow-up time were included. Based on a protocol of a 2-year interval grouping, included studies were categorized into four groups with follow-up periods  <24 months, 24–48 months, 48–72 months, and ≥72 months, respectively. Quality assessment was performed using the NOS score. Pooled estimations were computed by using a random-effects model in the STATA “metaprop” command. Publication bias was assessed visually through a funnel plot and statistically through Egger's linear regression test and Begg's correlation test.

**Results:**

A total of 44 studies were included containing 2,539 cases. Squamous metaplasia was the most commonly reported factor, followed by the extent of cyst removal. The other factors were reported individually. The pooled overall incidences of RCC recurrence after surgery in four groups of the follow-up period were 7.4% (95%CI = 4.1–11.3%) in <24 months, 13.1% (95%CI = 9.7–17.0%) in 24–48 months, 13.7% (95%CI = 7.7–21.0%) in 48–72 months, and 33.8% (95%CI = 19.6–49.6%) in ≥72 months. The pooled symptomatic incidences were 2.3% (95%CI = 0.4–5.1%) in <24 months, 5.6% (95%CI = 3.6–7.9%) in 24–48 months, 5.9% (95%CI = 2.4–10.6%) in 48–72 months, and 14.1% (95%CI = 6.0–24.5%) in ≥72 months. A dramatic increase in recurrent incidence was observed when the follow-up period was more than 72 months in both overall and symptomatic recurrence. A similar trend of recurrence was found in subgroup analyses stratified by publication year, cohort size, and cyst resection strategy.

**Conclusion:**

This study systematically reviewed recurrent factors and described the profile of trends in RCC recurrent incidence after surgery with a follow-up time based on a protocol of a 2-year interval, finding a dramatic increase in recurrent rates with a follow-up period of more than 72 months. This encouraged us to put forward a recommendation of at least a 6-year follow-up after surgery for patients with RCC.

**Systematic Review Registration:**

https://www.crd.york.ac.uk/prospero/, identifier: CRD42021278970.

## Introduction

Rathke's cleft cyst (RCC) is the most non-neoplastic lesion in the sellar region, typically located in the pars intermedia, between the adenohypophysis and the neurohypophysis (1). Although a majority of patients with RCC are asymptomatic and can be safely managed with serial MRI surveillance, symptoms may result in a compression of surrounding structures such as the pituitary gland, stalk, optic chiasma, and hypothalamus (2). Headache, visual impairment, and endocrine dysfunction are the most common clinical manifestations, while diabetes insipidus, aseptic meningitis, and mental disorder may also be found with the growth of RCC (3–5). When RCC is large enough to manifest as symptoms, surgery is recommended to aspirate cyst contents, thereby relieving the oppression of cyst (6). Successful decompression commonly results in the alleviation of symptoms, while the mass effect may recur with the recurrence of RCC. Despite the surgical treatment of RCC being exhaustively reported in previous studies over the past few decades, a wide range of incidences have occurred, which may be attributed to the existence of distinct protocols (7, 8). In addition, the potential association between recurrence and various factors such as squamous metaplasia in the cyst wall, suprasellar location, MRI rim enhancement of cyst, and the extent of resection has been put forward (2, 9). However, few studies have attempted any systematic summary of these diverse factors. We speculate that the wide range of follow-up time and different protocols may be the main reason for the obvious variation in recurrent incidence and factors. Therefore, we pooled the current literature to systematically review the recurrent factors and identify the incidence of RCC relapse in different follow-up periods after surgery. Furthermore, we attempted to explore an appropriate follow-up period by observing recurrent rates at different times.

## Methods

### Literature search strategy and selection criteria

This study was registered in PROSPERO (CRD42021278970) and conducted in accordance with Preferred Reporting Items for Systematic Review and Meta-Analysis (PRISMA) guidelines (10). The research question was designed using the PICOS format: “operated RCC patients” (Population), “radiographic follow-up” (Intervention), “different periods of follow-up” (Comparators), “incidence of RCC recurrence” (Outcomes), and “cases series studies” (Study designs). An electronic search in major public databases (Pubmed, Embase, Cochrane, and Web of Science) was conducted until September 12, 2022, using [rathke(Mesh) OR rathke OR rathke's] AND [recurrence(Mesh) OR recurrence OR recurrent OR relapse OR recur OR reaccumulation]. The details of the search strategies in these four public databases are summarized in Supplementary Table S1. Eligible studies should contain the following outcomes: (1) specific recurrent incidence after surgery and mean/median follow-up time; or (2) significant results of recurrent factors. In contrast, the exclusion criteria included studies with (1) languages other than English; (2) a pure pediatric cohort; (3) RCC in special locations or treated by special therapies (a pure suprasellar cohort, radiotherapy); (4) a lack of detailed information about recurrent incidence and follow-up time, as well as significant associated factors. Furthermore, studies involving multiple sellar lesions were excluded because their follow-up time was not specific for RCC. With regard to publication type, full-text articles were required, while reviews, case reports, commentaries, letters, and editorials were excluded. The reference lists of included studies were hand-searched to identify potentially relevant studies. In addition, for overlapping cohorts reported from the same institutions, only complete studies were included. The literature search and selection were performed independently by two reviewers (AQ and ZJ) using Endnote X9, and any discrepancies were discussed and resolved by consultation with the third reviewer (XSW).

### Data extraction

For eligible studies, the following information was extracted: author name, year of publication, study country, study design, demographic characteristics (cohort size, sex, and mean years), number of recurrences, type of surgery, cyst resection strategy, follow-up time, and recurrent factors (if available). The definition of recurrence was the radiological cyst regrowth or progression of residual cyst. Cases without any cyst residual or with aggressive cyst resection to pursue radical excision were categorized as gross total resection (GTR). Those with residual cyst, including partial resection, decompression, biopsy, fenestration, and marsupialization, or conservative resection for functional preservation were defined as subtotal resection (STR) (9).

### Quality assessment

The Newcastle–Ottawa Scale (NOS) was adopted for quality assessment, which was recommended by the Cochrane Nonrandomized Studies Methods Working Group (11). A detailed description of this scale has been reported in a previous study (12). The quality assessment was performed by two independent reviewers (XZ and JJY), and any discrepancies were figured out through discussion with the third reviewer (XSW).

### Meta-Analysis

In the current study, only studies containing a pure RCC cohort with specific incidence of recurrence and follow-up time were included for quantitative estimation. Considering the number of studies in each follow-up period, the follow-up times were grouped based on a protocol of 2-year intervals, that is, groups with follow-up periods <24 months, 24–48 months (excluding 48 months), 48–72 months (excluding 72 months), and ≥72 months. The *I*^2^ and Cochran's Q homogeneity tests were performed to evaluate the statistical heterogeneity. The recurrent incidence and 95% confidence intervals (CI) were calculated from each study. The weight assigned to each study and estimated pooled rate in forest plots were computed by running the “metaprop” command in STATA with a random effects model. The variances before pooling were stabilized by performing the Freeman–Turkey double arcsine transformation (13). Subgroup analyses were performed based on publication year (before 2015, or 2015+), cohort size (≥40 cases, or <40 cases), and cyst resection strategy (either STR or GTR). Sensitive analysis was performed by adopting the leave-one-out approach to determine if there were any deviations in the pooled estimation (12). If the number of studies was more than 10, potential publication bias was assessed by observing symmetry in a funnel plot and using statistical results obtained from Egger's linear regression test and Begg's correlation test (14, 15). All data analyses were performed with STATA 15.1.

## Results

### Characteristics of included studies

**Figure 1:**
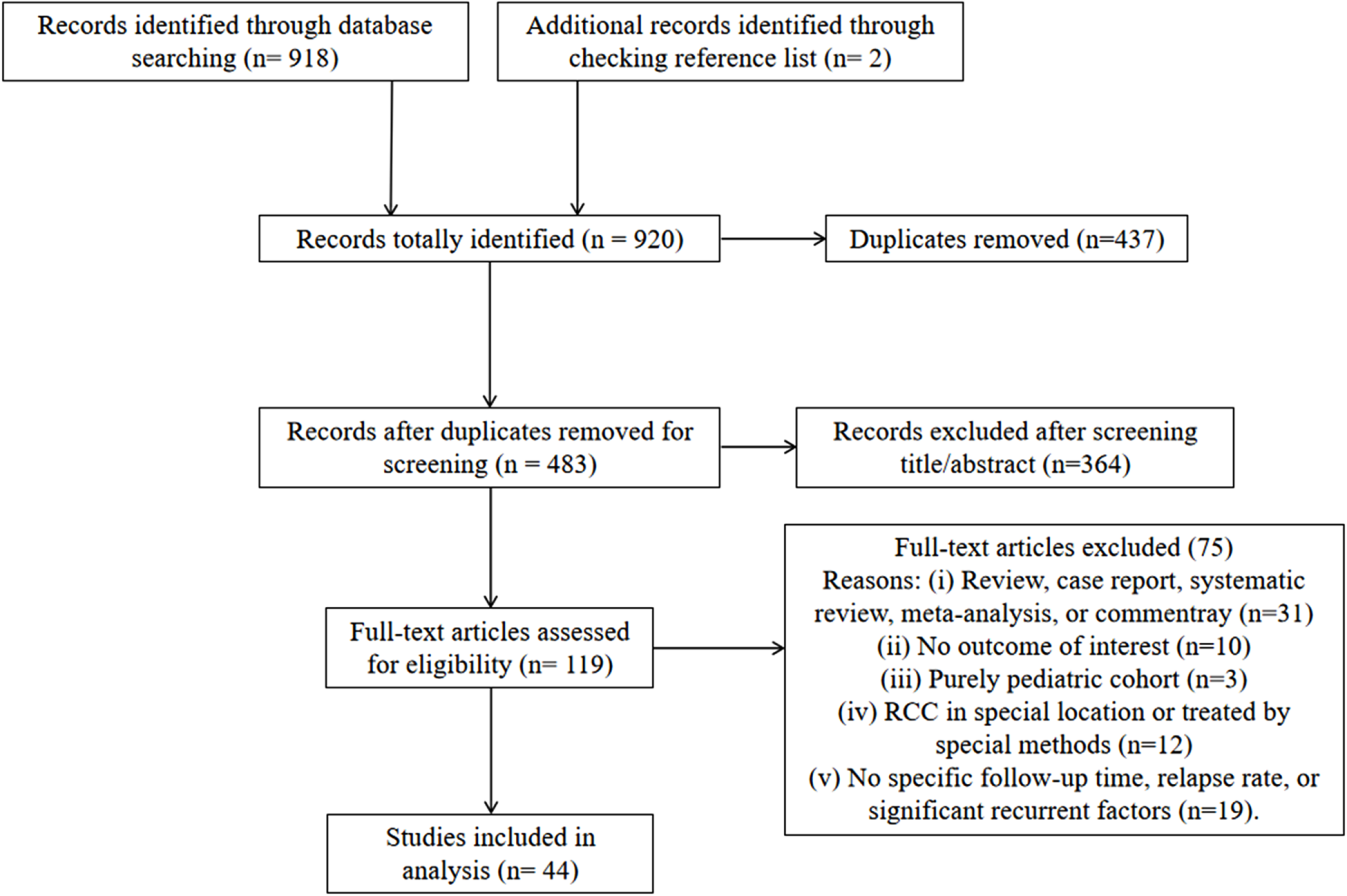
Process of study selection based on a PRISMA search strategy.

The detailed process of literature search and selection is shown in Figure 1. In total, 920 studies were initially identified. After excluding 437 duplications and 364 articles from title/abstract screening, 119 studies were included for full-text assessment. Finally, 44 retrospective studies were qualified for this analysis, including 2,539 cases with a mean/median follow-up time ranging from 15 to 96 months (Table 1). Forty-two studies reported detailed recurrent incidences and mean/median follow-up times. Specifically, based on a 2-year interval grouping, 8 studies with a follow-up period < 24 months (16–23), 16 studies within 24–48 months (8, 24–38), 12 studies within 48–72 months (2, 4, 7, 39–47), and 6 studies ≥ 72 months (48–53) were categorized. The rates of overall recurrence and those with symptoms in individual studies were in the range of 0%–60% and 0%–39.1%, respectively. The total score of quality rating in each study ranged from 5 to 8 with an average of 6.1, indicating the moderate quality of the included studies (Table 1).

**Table 1:** Demographics and characteristics of included studies.

Authors (year)	Country	Study design	Cohort size	Sex		Age (year)	Recurrence (%)	Type of surgery	Resection strategies	Follow-up (months)	NOS score
				Female	Male						
Aho (2005)	USA	Retro-	118	68	50	NR	17.8	mTSS	GTR + STR	NR	7
Arko (2021)	USA	Retro-	31	23	8	NR	25.8	eTSS	STR	28	6
Barkhoudarian (2019)	USA	Retro-	36	29	7	38	25.0	eTSS	STR	24	5
Benveniste (2004)	USA	Retro-	62	49	13	NR	16.1	mTSS + eTSS	GTR + STR	28	6
Cabuk (2019)	Turkey	Retro-	84	67	17	33	2.4	eTSS	GTR + STR	59	7
Chong (2022)	China	Retro-	37	23	14	48	59.5	NR	NR	8.1 years	5
Chotai (2015)	China	Retro-	87	64	23	41	12.6	eTSS + TCS	GTR + STR	54	7
Cohan (2004)	USA	Retro-	24	19	5	31	8.3	mTSS	GTR + STR	17	5
El-Mahdy (1998)	UK	Retro-	28	16	12	45	3.6	mTSS	GTR + STR	24	5
Fan (2012)	China	Retro-	42	23	19	42	7.1	mTSS + TCS	GTR + STR	22	5
Frank (2005)	Italy	Retro-	22	14	8	37	4.5	eTSS	GTR + STR	33	5
Higgins (2011)	USA	Retro-	61	45	16	43	18.0	TSS^a^+ TCS	GTR + STR	61	8
Jiang (2018)	China	Retro-	13	8	5	45	0.0	eTSS	STR	17	5
Kasperbauer (2002)	USA	Retro-	29	18	11	46	27.6	mTSS + eTSS + TCS	NR	6.6 years	6
Kim (2004)	Korea	Retro-	53	28	25	37	11.3	mTSS + TCS	GTR + STR	31	7
Kim (2012)	Korea	Retro-	40	29	11	41	10.0	mTSS + eTSS + TCS	STR	76	7
Kinoshita (2016)	Japan	Retro-	91	55	36	53	39.6	mTSS + eTSS + TCS	STR	80	8
Koutourousiou (2009)	Greece	Retro-	14	10	4	44	14.3	mTSS	GTR	29	5
Kuan (2017)	USA	Retro-	52	31	21	41	9.6	eTSS	STR	20	6
Langlois (2019)	USA	Retro-	73	56	17	40	24.7	TSS^a^	GTR + STR	4.0 years	7
Lillehei (2011)	USA	Retro-	82	57	25	37	10.7	TSS^a^+ TCS	STR	46	6
Limb (2021)	Australia	Retro-	23	NR	NR	NR	47.8	mTSS + eTSS + TCS	NR	96	5
Lin (2019)	USA	Retro-	109	80	29	45	26.6	mTSS + eTSS	GTR + STR	67	7
Madhok (2010)	USA	Retro-	35	NR	NR	34	5.7	eTSS	STR	19	6
Marcus (2020)	UK	Retro-	61	F:M = 2:1		55	19.7	mTSS + eTSS	GTR + STR	34	7
Mendelson (2015)	USA	Retro-	11	5	6	43	36.4	eTSS	STR	24	5
Montaser (2021)	USA	Retro-	116	86	30	39	11.2	eTSS	STR	15	6
Mukherjee (1997)	UK	Retro-	12	6	6	30	33.3	mTSS + TCS	STR	30	5
Ogawa (2011)	Japan	Retro-	155	97	58	46	17.4	TSS^a^	STR	24	7
Park (2012)	USA	Retro-	73	40	33	35	16.4	mTSS	STR	59	7
Potts (2011)	USA	Retro-	151	119	32	40	11.3	mTSS + eTSS	STR	30	8
Ratha (2017)	India	Retro-	27	17	10	NR	3.7	mTSS + eTSS	GTR + STR	38	5
Ross (1992)	USA	Retro-	43	33	10	34	2.3	mTSS + TCS	STR	68	6
Sade (2005)	Canada	Retro-	10	6	4	40	0.0	TSS^a^+TCS	GTR + STR	32	5
Sala (2018)	USA	Retro-	72	53	19	45	13.9	TSS^a^	GTR + STR	54	6
Sharifi (2022)	Iran	Retro-	27	24	3	38	0.0	eTSS + TCS	GTR + STR	18	5
Tate (2010)	USA	Retro-	176	132	44	NR	11.4	mTSS + TCS	STR	NR	6
Trifanescu (2011)	UK	Retro-	33	20	13	43	21.9	TSS^a^+ TCS	STR	48	6
Truong (2021)	France	Retro-	18	14	4	36.7	23.5	mTSS + eTSS + TCS	GTR + STR	73	5
Wait (2010)	USA	Retro-	73	56	17	40	11.0	mTSS	GTR + STR	27	7
Wedemeyer (2019)	USA	Retro-	91	68	23	42	21.8	mTSS + eTSS + TCS	STR	69	7
Yamada (2022)	Japan	Retro-	27	20	7	45	29.6	eTSS	STR	52	6
Zhang (2021)	China	Retro-	72	42	30	42	0.0	eTSS	GTR + STR	61	7
Zhong (2012)	China	Retro-	45	24	21	47	14.6	mTSS + TCS	GTR + STR	16	6

NR, no record; eTSS, endoscopic transsphenoidal surgery; mTSS, microscopic transsphenoidal surgery; TCS, transcranial surgery; GTR, gross total resection; STR, subtotal resection.

^a^ Not clarified endoscopic or microscopic.

### Systematic review of recurrent factors

In total, 14 studies reported the significant results of recurrent factors (Table 2). Seven studies found a significant association between RCC recurrence and squamous metaplasia of the cyst wall (2, 26, 29, 37, 41, 51, 54). Four studies suggested a higher incidence of recurrence in patients with a residual cyst wall (29, 41, 43, 45). As for preoperative demographic and clinical characteristics, Montaser et al. (21) demonstrated that RCC recurrence was more prone in female patients (female vs. male = 15% vs. 0). Wedemeyer et al. (45) found that patients with more than 40 years of age were more likely to experience RCC relapse (≥40 vs. <40 years = 34.4% vs. 15.6%). Langlois et al. (40) observed less-frequent headache cases in recurrent vs. non-recurrent RCC (72% vs. 89%), while the proportion was high in both groups. Moreover, Sala et al. (43) detected an association between increased recurrence and preoperative hormone disorder (growth hormone deficiency and hyperprolactinemia). As for the predictive efficacy of preoperative MRI to relapse, Chotai et al. (2) identified the independent recurrent risk factors of isointensity on T2-weighted imaging (OR 7.7, 95% CI = 1.75–34.54) and suprasellar RCC (OR 10.29, 95% CI = 1.094–96.872). However, Potts et al. (35) put forward the conclusion that compared with the intrasellar subtype, both intrasuprasellar and suprasellar RCC (RCC with a suprasellar component) had a significant association with recurrence (HR 2.6, 95% CI = 1.8–4.1). Ogawa et al. (34) suggested that the factor of cyst with cerebrospinal fluid (CSF)-like intensity on T1-weighted imaging accelerated RCC reaccumulation. Pathologically, besides squamous metaplasia, Benveniste et al. (26) found that cyst inflammation was correlated with an increased risk of relapse (31.6% vs. 9.8%). Similarly, Tate et al. (55) identified the independent recurrent predictor of RCC infection (HR 4.5, 95% CI = 1.1–18.6), while a non-significant association was found between inflammation and recurrence. Despite various factors of RCC recurrence being put forward by many studies, only four studies clarified adjusted outcomes based on multivariate analysis (2, 29, 35, 55), which limited the disclosure of intrinsic risk factors by quantitative estimation.

**Table 2:** Summary of reported recurrent factors in previous studies.

Study (year)	Factors
Aho (2005)	Graft packing, squamous metaplasia
Benveniste (2004)	Squamous metaplasia, inflammation
Chotai (2015)	Suprasellar RCC, isointensity on T2, squamous metaplasia
Kim (2004)	Enhancement on MRI, extent of removal, squamous metaplasia
Kinoshita (2016)	Squamous metaplasia
Langlois (2019)	Headache
Lin (2019)	Extent of removal, squamous metaplasia
Montaser (2021)	Female patients
Ogawa (2011)	CSF-like intensity, epithelial transition
Potts (2011)	Suprasellar component
Sala (2018)	Growth hormone deficiency, hyperprolactinemia, extent of removal; CSF leakage
Tate (2010)	RCC infection
Wait (2010)	Squamous metaplasia
Wedemeyer (2019)	Age ≥40, extent of removal

RCC, Rathke's cleft cyst; CSF, cerebrospinal fluid.

### Meta-analysis of recurrent incidence

**Figure 2:**
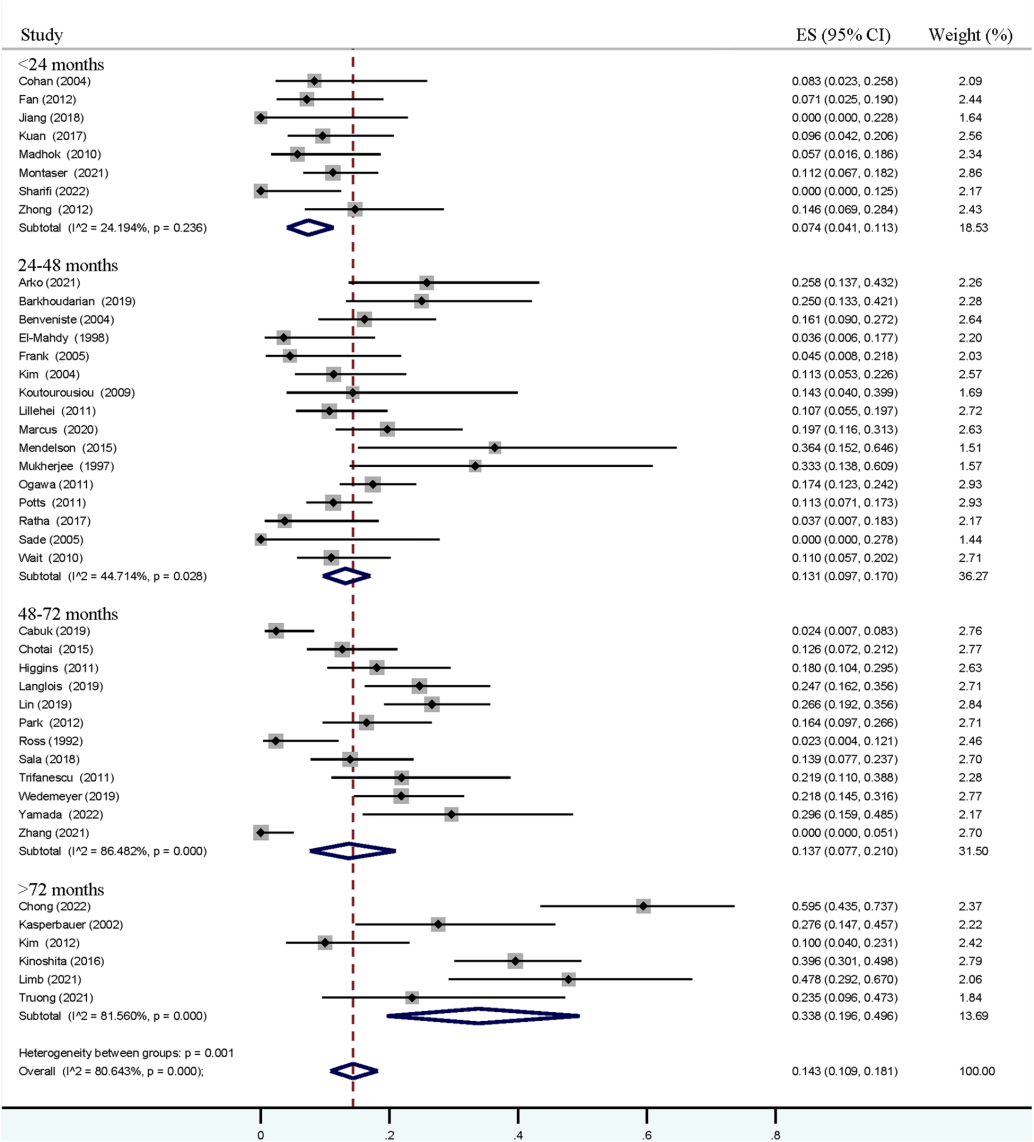
Forest plot demonstrating pooled estimations of overall recurrent incidence of Rathke's cleft cyst in groups with follow-up periods <24 months, 24–48 months, 48–72 months, and ≥72 months. Obvious heterogeneity (*I^2^* > 40%) was found in all groups, expect in those with follow-up period <24 months.

**Figure 3:**
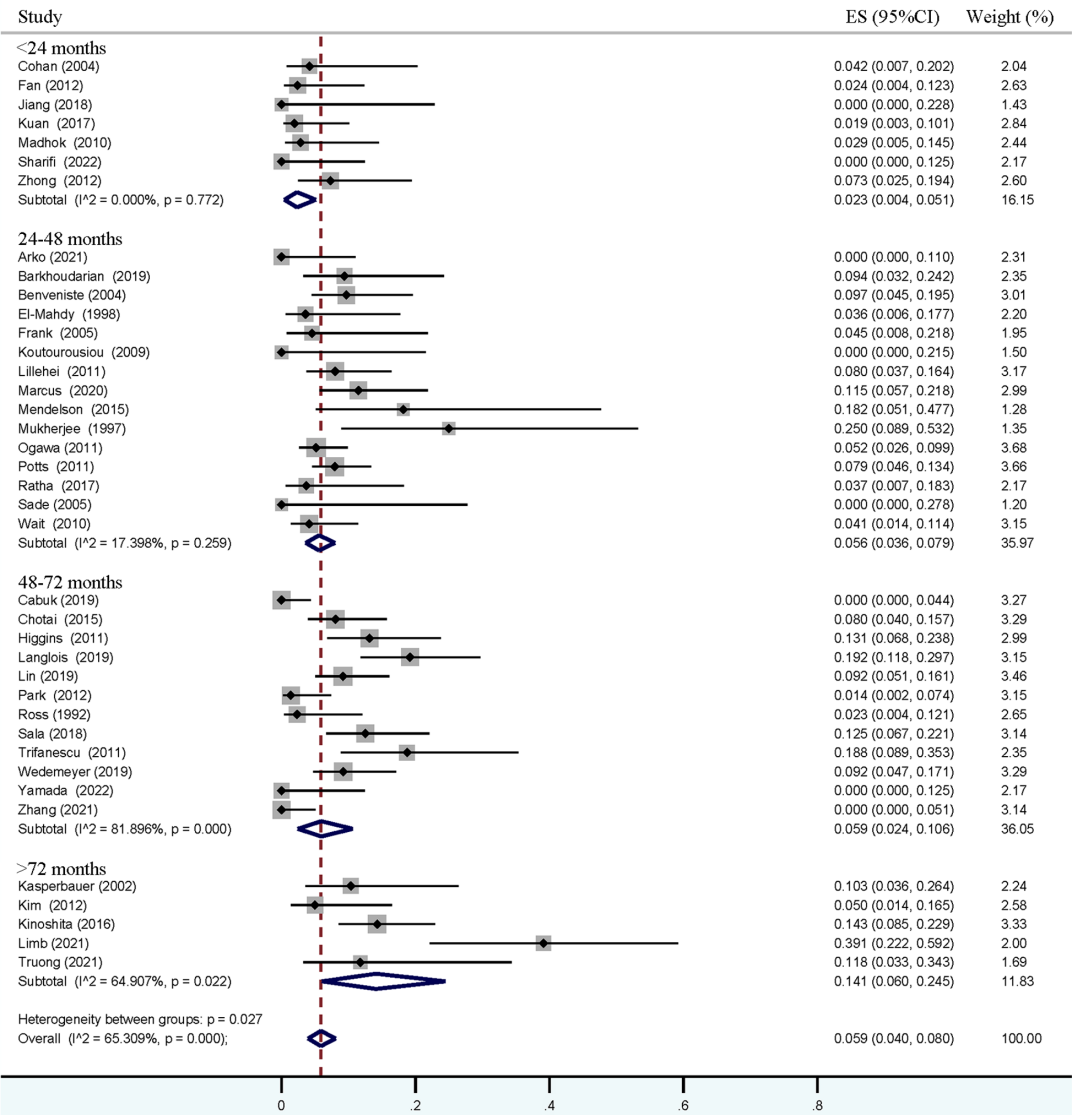
Forest plot showing pooled estimations of symptomatic recurrent incidence of Rathke's cleft cyst in groups with follow-up periods <24 months, 24–48 months, 48–72 months, and ≥72 months. Obvious heterogeneity (*I^2^* > 40%) was found in groups with follow-up period ≥ 48 months.

As shown in Figure 2, except the analysis in the <24-month group, a relatively high heterogeneity was found in each pooled estimation. The pooled overall incidence of recurrence was positively associated with the follow-up time, revealing incidence from 7.4% (95% CI = 4.1%–11.3%) to 33.8% (95% CI = 19.6%–49.6%). Similar pooled rates were noticed in follow-up times within 24–48 months (13.1%, 95% CI = 9.7%–17.0%) and 48–72 months (13.7%, 95% CI = 7.7%–21.0%). A total of 39 of the 42 studies specifically reported the incidence of symptomatic recurrence, and a comparatively high heterogeneity was also observed in groups with a follow-up time of more than 48 months (Figure 3). An increased rate trend was also found in pooled symptomatic recurrence, which ranged from 2.3% (95% CI = 0.4%–5.1%) in the follow-up period < 24 months to 14.1% (95% CI = 6.0%–24.5%) in the period ≥ 72 months. Meanwhile, similar pooled incidences of symptomatic relapse were found in follow-up periods within 24–48 months (5.6%, 95% CI = 3.6%–7.9%) and 48–72 months (5.9%, 95% CI = 2.4%–10.6%).

### Subgroup analyses of overall recurrent incidence

**Figure 4:**
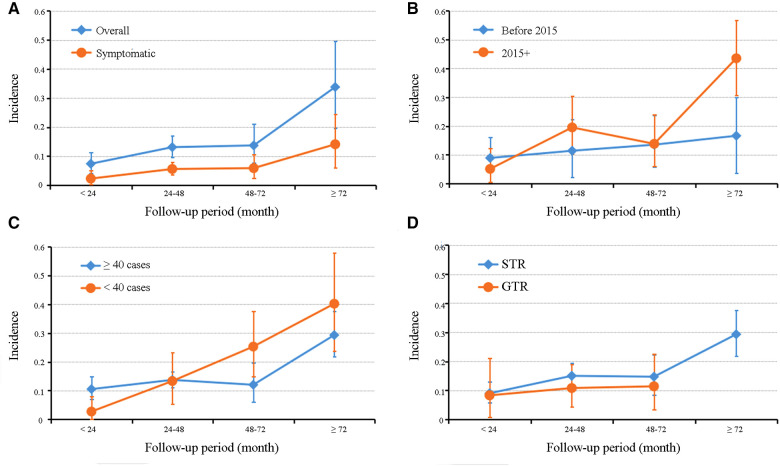
Graphs of a line chart showing the trend of recurrent incidence of Rathke's cleft cyst in overall and symptomatic pooled estimations (**A**) and subgroup analyses based on publication year (**B**), cohort size (**C**), and cyst resection strategy (**D**).

As summarized in Table 3, subgroup analyses stratified by publication years, cohort size, and cyst resection strategy were performed to explore pooled overall incidence in classified follow-up periods under different conditions. The grouped rates of relapse were found in the range of 2.7%–43.5%; however, high heterogeneity was also present in most subgroups. The line charts in Figure 4 demonstrated that most subgroups experienced a stable fluctuation of recurrent incidence with a follow-up period of less than 72 months, while the steeply upward trend occurred after 72 months of follow-up. Unfortunately, there was no report of RCC undergoing GTR with a follow-up period of more than 72 months in these included studies.

**Table 3:** Subgroup analyses of the overall incidence of RCC recurrence.

	Number of studies	*I*-square (%)	Pooled event rate with 95%CI
Published year			
Before 2015			
Follow-up <24 months	4	72.5	0.089 (0.044, 0.144)
24–48 months	11	85.4	0.114 (0.084, 0.148)
48–72 months	4	96.4	0.135 (0.058, 0.236)
≥72 months	2	91.1	0.166 (0.084,0.265)
2015+			
Follow-up <24 months	4	0.0	0.051 (0.006, 0.123)
24–48 months	5	91.9	0.195 (0.103, 0.305)
48–72 months	8	97.2	0.138 (0.060, 0.239)
≥72 months	4	73.9	0.435 (0.306, 0.568)
Cohort size			
≥40 cases			
Follow-up <24 months	4	56.2	0.105 (0.069, 0.148)
24–48 months	7	72.3	0.137 (0.111, 0.166)
48–72 months	10	88.3	0.120 (0.060, 0.197)
≥72 months	2	94.4	0.293 (0.217, 0.375)
<40 cases			
Follow-up <24 months	4	11.2	0.027 (0.000, 0.080)
24–48 months	9	67.3	0.133 (0.054, 0.232)
48–72 months	2	3.3	0.253 (0.148, 0.375)
≥72 months	4	73.1	0.402 (0.236, 0.579)
Resection strategy			
STR			
Follow-up <24 months	6	72.0	0.090 (0.057, 0.129)
24–48 months	15	83.9	0.150 (0.110, 0.193)
48–72 months	11	95.5	0.147 (0.085, 0.222)
≥72 months	3	97.4	0.293 (0.217, 0.375)
GTR			
Follow-up <24 months	2	0.0	0.083 (0.007, 0.210)
24–48 months	3	46.8	0.108 (0.044, 0.189)
48–72 months	5	78.9	0.114 (0.034, 0.225)

STR, subtotal resection; GTR, gross total resection.

### Sensitive analysis and publication bias assessment

**Figure 5:**
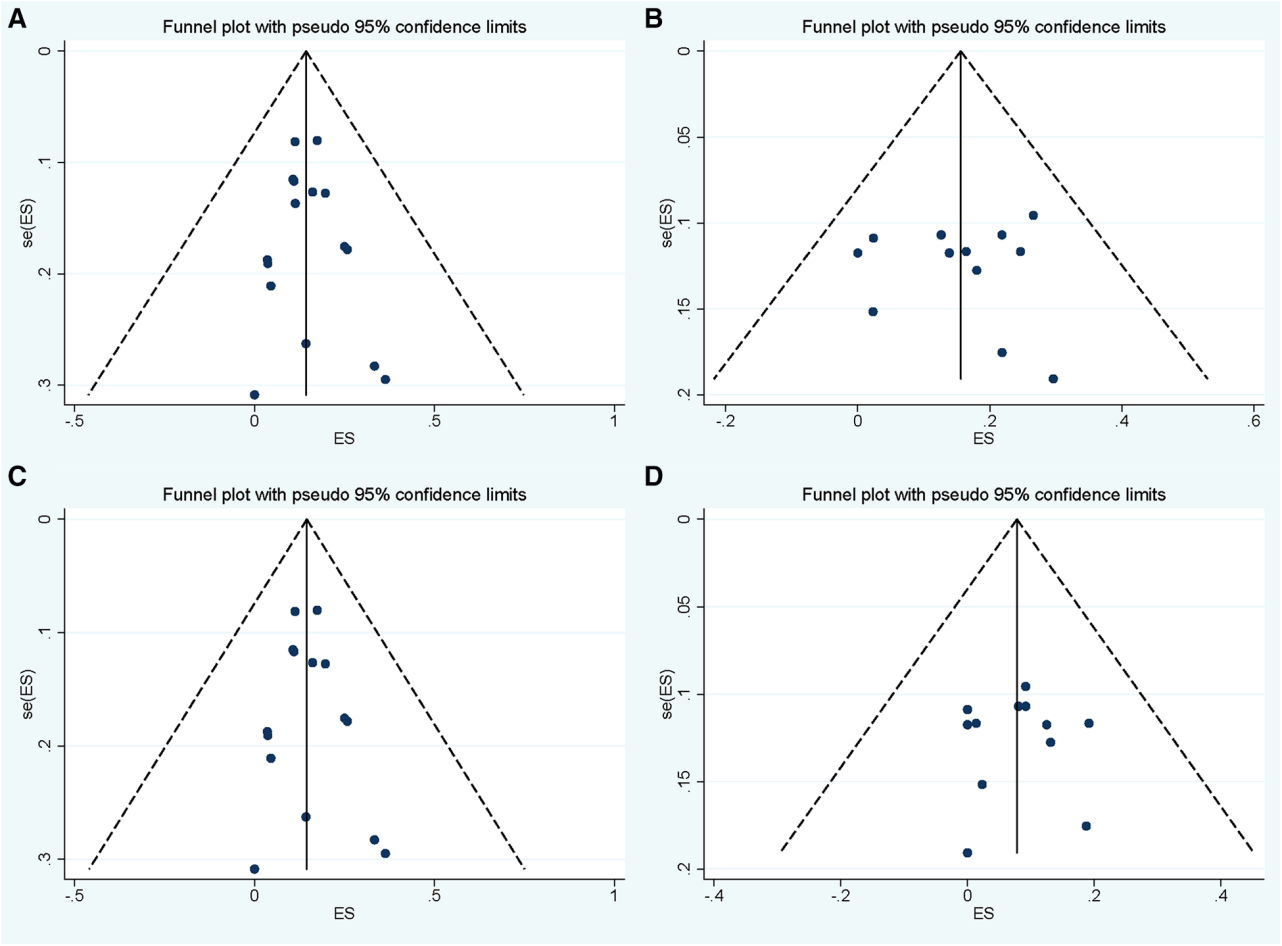
Funnel plot for publication bias assessment of pooled overall and symptomatic recurrent incidence of Rathke's cleft cyst in groups with follow-up period within 24–48 months (**A,C**) and 48–72 months (**B,D**).

No significance was found in sensitive analysis when meta-analysis was repeated following the exclusion of each study (Supplementary Figure S1). Publication bias assessment was performed in groups of overall and symptomatic recurrence with follow-up periods within 24–48 months and 48–72 months. Slight asymmetries were found in these funnel plots (Figure 5), while both Egger's linear regression test and Begg's correlation test showed no significance in all these four pooled analyses (all *p* > 0.1). The other groups of follow-up periods (<24 or ≥72 months) in the pooled analyses of overall and symptomatic recurrence were not subjected to bias assessment because of the limited number of enrolled studies.

## Discussion

RCC mostly presents as a benign lesion with good postoperative recovery; however, the potential tendency of recurrence makes postoperative monitoring a thorny problem. Although small radiological recurrence can still be tracked with MRI surveillance, surgical decompression is commonly required for patients with symptomatic relapse (4). Numerous authors have reported their follow-up results in the surgical management of RCC and put forward a wide range of recurrent incidences and various associated factors (19, 30, 37, 40, 41, 47, 48). The wide variation in follow-up times may be the primary reason for these inconclusive results, which also rendered the rate of a simple pooled estimation inaccurate. Clarifying the real recurrent incidence and factors of RCC is crucial for formulating the most appropriate follow-up protocols for individual patients. Therefore, we first performed a meta-analysis of RCC recurrent incidence based on the follow-up period to establish a clearer picture of RCC recurrence.

In this study, we found that the pooled recurrent incidences of RCC were 7.4%–33.8% with the follow-up period ranging from <24 months to ≥72 months and also observed their positive association with the follow-up time. Critically, we may indirectly resolve the controversy over the recommended follow-up period. The positive association between RCC recurrence after surgery and follow-up time is widely accepted. Ogawa et al. suggested that follow-up MR imaging should be performed at least 36 months after surgery, even if no reaccumulation was detected (56). However, Langlois et al. recommended at least a 5-year follow-up protocol in their large single-center study (40). Kim et al. even put forward a recommendation of at least a 10-year follow-up after surgery (50). However, recommendations with too long follow-up periods not only increase the economic pressure of patients, but also aggravate their psychological burden, probably resulting in loss of follow-up about a decade after surgery. Meanwhile, excessive radiographic exposure may increase the risk of neoplastic diseases such as glioma (57). An appropriately prolonged follow-up is necessary to the extent that potential recurrence can be detected under continuous radiographic examination. For patients with recurrent asymptomatic RCC, the follow-up of imaging, ophthalmic, and endocrine examination should be strengthened with hormone supplements (if deficient), and surgical decompression is recommended when symptoms or a tendency of cyst enlargement occur (50). In this study, the rates of both overall and symptomatic recurrences were stable before 72 months of follow-up (groups of follow-up <24 months, 24–48 months, and 48–72 months), while a dramatic increase in the recurrent rate was observed after 72 months. These results suggested that, in our 2-year interval grouping, obvious recurrence would be detected with a follow-up of at least 6 years; that is, the recommendation of the follow-up time should be at least 6 years. Unfortunately, a limited number of studies at each follow-up period prevents us from determining the profile of RCC recurrent trends from more detailed groupings (e.g., 1-year intervals). In addition, only two of the included studies possess a follow-up time of more than 8 years (96 months), which places limitations on us to explore the trends in recurrent rate with a follow-up of more than 6 years.

Similar relationships between recurrent incidence and follow-up period were found in most subgroup analyses. Cohort size is regarded as another vital factor impacting the observation of real recurrent incidence (2). Subgroup analyses based on cohort size were performed, demonstrating the same trends in the group with a large sample size (≥40 cases) as the overall recurrent rate. We observed that in the follow-up period ≥ 24 months, higher rates of relapse were found in the small sample–size group, compared with the large sample–size group. This might possibly be attributed to the fact that each case in the cohort with small size accounted for a comparatively large proportion, which allowed each case of recurrence to cause a greater impact on incidence and decreased the predictive value for recurrence.

Various risk factors of RCC recurrence after surgery have been systematically reviewed in this study, demonstrating the squamous metaplasia of the cyst wall as the most reported factor, followed by the extent of cyst resection. However, other predictors that may theoretically increase RCC recurrence have been reported only individually, such as graft packing and cyst inflammation (26, 54). As a strong predictor of RCC relapse, Sade et al. (38) found that RCC with predominant squamous metaplasia was shown to possess a higher proliferation index. One theory indicates that RCC and craniopharyngioma may be two poles of the same disease spectrum, and squamous metaplasia may be an etiology stimulating RCC to transform into papillary craniopharyngioma (2, 34). In addition, some significant factors associated with RCC recurrence after surgery serve as a stimulator or characteristic of squamous metaplasia, such as cyst inflammation and rim enhancement on MRI (26, 29). Chotai et al. (2) suggested that suprasellar RCC was not only a risk factor for relapse, but also an independent predictor of squamous metaplasia. To sum up, we state that squamous metaplasia may play an important role in RCC recurrence after surgery, but more advanced evidence-based studies are still required to prove our contention.

To the best of our knowledge, only the predictive value of the extent of cyst resection has been proven by a meta-analysis (9). Lu et al. conducted a meta-analysis to compare the effect of the extent of cyst resection on RCC recurrence, finding a significantly decreased risk of recurrence in those undergoing GTR rather than STR (9). Similar results were also found in this study. Subgroup analyses based on the extent of cyst resection demonstrated a higher incidence of RCC recurrence in the STR group compared with that in the GTR group in the same follow-up period. Moreover, a dramatic increase in recurrent rate was also observed in the STR group with a follow-up of more than 72 months. Unfortunately, to date, there has been no study evaluating the recurrence of patients undergoing GTR with a specific mean/median follow-up time of more than 72 months. Therefore, we suggest that the residual cyst wall is the main source of RCC recurrence. In contrast, Mendelson et al. (58) performed a meta-analysis in 2013 to evaluate the pooled recurrent rate of RCC after surgery, reporting that the weighted average of incidence in GTR was 19%, in STR it was 9%, and in drainage or biopsy it was 6%. Furthermore, for surgical techniques, the weighted average of incidence after microscopic transsphenoidal surgery (mTSS) was 14%, and for endoscopic surgery, it was 8%. In fact, in most meta-analyses, studies on cyst resection are performed microscopically, while more than half of the studies enrolled (24/44) in the present meta-analysis have adopted endoscopic transsphenoidal surgery. Even if GTR is described, cyst resection under the endoscope is a more radical procedure with a clearer visualization compared with that under the microscope, resulting in lower recurrent incidence (9). In addition, the follow-up period of the studies included by Mendelson et al. was short and limited within a narrow range to the extent that the obtained rate may reflect the recurrence of RCC only in a particular period (58), both of which may contribute to the contradiction between these two studies. However, detailed information on recurrence in different surgical methods was not available in a large proportion of included studies, which prevented us from performing subgroup analysis based on microscopic and endoscopic transsphenoidal surgery.

Despite the association between GTR and decreased incidence of RCC, recurrence has been identified. Lu et al. also suggested an elevated risk of postoperative diabetes insipidus in those with GTR, in their meta-analysis (9). Furthermore, from the perspective of electrolyte, our previous study found a significantly higher risk of postoperative hyponatremia in patients with an excessive resection of the cyst wall (59). Although in this meta-analysis, we found that the STR subgroup possessed a slightly higher incidence of RCC recurrence than the GTR subgroup at each follow-up period, we still felt that as a benign lesion with great prognosis, priority should be given to the functional preservation of surrounding structures during the surgical treatment of RCC, instead of excessive cyst resection.

Certainly, our study has several limitations. First, a relatively high heterogeneity was present in pooled estimations, which may be ascribed to the study design and the varied sample size. Even if study stratification and sensitive analysis were adopted, no specific source of heterogeneity could be indicated. Second, the low number of eligible studies in different follow-up periods prevented us from exploring the trend of recurrence in more detailed groupings (e.g., 1-year intervals). Subgroup analyses further reduced the sample size, that may have rendered the corresponding results ineffective. Third, there was a large proportion of studies with small cohort sizes, which may have impacted the real trends of recurrent incidence. Fourth, few studies have reported the adjusted results of recurrent factors based on multivariate analysis, which placed limitations on us in confirming the real predictors by quantitative estimation. Therefore, we suggest that a more comprehensive meta-analysis with sufficient eligible studies would be able to provide a more accurate incidence of recurrence in different follow-up periods and the various associated factors in future.

## Conclusion

This study systematically reviewed the factors associated with RCC recurrence and quantitatively evaluated the incidences of RCC recurrence in different follow-up periods, which, however, proved difficult to produce a pooled estimate due to a wide variation of follow-up times. Meanwhile, the rates of symptomatic recurrence in different follow-up periods were also presented. Despite the existence of several limitations, this study can partly help surgeons to understand the recurrent factors and the profile of trends in RCC recurrent incidence with follow-up times. Critically, based on a 2-year interval grouping, we found a dramatic increase in recurrent incidence with a follow-up period ≥ 72 months, as a result of which we put forward a recommendation of at least a 6-year follow-up after surgery for patients with RCC.

## Data Availability

The original contributions presented in the study are included in the article/**Supplementary Material**, and further inquiries can be directed to the corresponding author/s.
