# Supplementary figures and images for: Incidence and factors associated with the recurrence of Rathke's cleft cyst after surgery: A systematic review and meta-analysis
Source: Front Surg. 2023 Jan 5;9:1065316. doi: 10.3389/fsurg.2022.1065316 (PMC9849585; doi:10.3389/fsurg.2022.1065316)

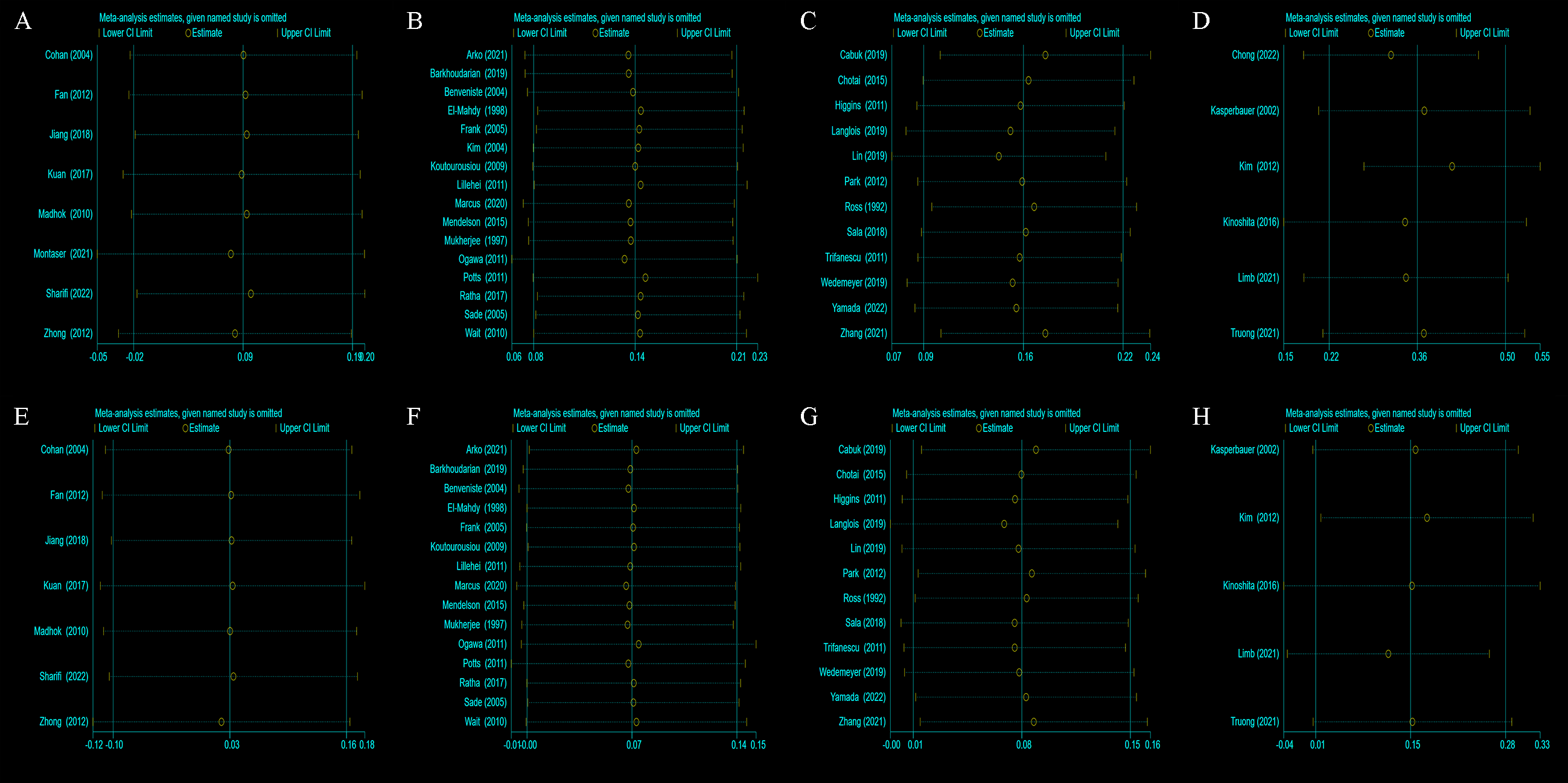

Supplement: Supplementary file 1 [file Image1.tif]
