# Supplementary material for: Incidence and factors associated with the recurrence of Rathke's cleft cyst after surgery: A systematic review and meta-analysis
Source: Front Surg. 2023 Jan 5;9:1065316. doi: 10.3389/fsurg.2022.1065316 (PMC9849585; doi:10.3389/fsurg.2022.1065316)
Supplement: Supplementary file 2 [file Table1.doc]

Supplementary Table 1. Search strategies in four public databases.

| Public databases | Search strategies | Number of studies |
| --- | --- | --- |
| Pubmed | (rathke[Mesh] OR rathke OR rathke's) AND (recurrence[Mesh] OR recurrence OR recurrent OR relapse OR recur OR re-accumulation) | 234 |
| Embase | (('rathke cleft cyst'/exp) OR ('rathke')) AND (('relapse'/exp) OR ('recurrence' ) OR ('recurrent') OR ('recurrent') OR ('relapse') OR ('recur') OR ('re-accumulation')) | 444 |
| Cochrane | (rathke) OR (rathke’s) | 17 |
| Web of science | (TS= (rathke) or ALL=(rathke OR rathke's)) AND (TS= (recurrence) OR ALL=(recurrence OR recurrent OR relapse OR recur OR re-accumulation)) | 223 |
